# Saquinavir plus methylprednisolone ameliorates experimental acute lung injury

**DOI:** 10.1590/1414-431X20187579

**Published:** 2018-08-06

**Authors:** Guanghua Zhang, Xue Zhang, Haidi Huang, Yunxia Ji, Defang Li, Wanglin Jiang

**Affiliations:** School of Pharmacy, Binzhou Medical University, Yantai, China

**Keywords:** Saquinavir, ALI, Two-hit, HMGB1, TLR4

## Abstract

Glucocorticoid insensitivity is an important barrier to the treatment of several inflammatory diseases, including acute lung injury (ALI). Saquinavir (SQV) is an inhibitor of the human immunodeficiency virus protease, and the therapeutic effects of SQV in ALI accompanied with glucocorticoid insensitivity have not been previously investigated. In this study, the effects of SQV on lipopolysaccharide (LPS)-mediated injury in human pulmonary microvascular endothelial cells (HPMECs), human type I alveolar epithelial cells (AT I), and alveolar macrophages were determined. In addition, the effects of SQV on an LPS-induced ALI model with or without methylprednisolone (MPS) were studied. In LPS-stimulated HPMECs, SQV treatment resulted in a decrease of high mobility group box 1 (HMGB1), phospho-NF-κB (p-NF-κB), and toll-like receptor 4 (TLR4), and an increase of VE-cadherin. Compared to MPS alone, MPS plus SQV attenuated the decrease of glucocorticoid receptor alpha (GRα) and IκBα in LPS-stimulated HPMECs. HMGB1, TLR4, and p-NF-κB expression were also lessened in LPS-stimulated alveolar macrophages with SQV treatment. In addition, SQV reduced the injury in human AT I with a decrease of HMGB1 and p-NF-κB, and with an increase of aquaporin 5 (AQP 5). SQV ameliorated the lung injury caused by LPS in rats with reductions in vascular permeability, myeloperoxidase activity (MPO) and histopathological scores, and with lowered HMGB1, TLR4, and p-NF-κB expression, but with enhanced VE-cadherin expression. By comparison, SQV plus MPS increased GRα and IκBα in lung tissues of rats with ALI. This study demonstrated that SQV prevented experimental ALI and improved glucocorticoid insensitivity by modulating the HMGB1/TLR4 pathway.

## Introduction

Acute respiratory distress syndrome (ARDS) is characterized by acute onset, hypoxia, and diffuse infiltrates on chest X-rays. The lungs of ARDS patients are heavy with atelectasis, interstitial and alveolar edema, and hyaline membranes (1), and the overall mortality rate is 34.9–46.1% (2). The pathogenesis of ARDS is the result of direct insults on lung cells and indirect insults as a result of acute systemic inflammatory responses that are key factors in determining the development and progression of acute lung injury (ALI) (3).

An essential early step in the inflammatory reaction of ALI and ARDS is the recruitment of macrophages, followed by their adherence to the vascular endothelium to permeate vessel walls (pulmonary microvascular endothelial cells) and to penetrate tissues. Accumulated leukocytes in the injured lungs further lead to increases in proinflammatory cytokines and adhesion molecules (4). Thus, it is urgent to find effective drugs to inhibit inflammation and improve ARDS therapy.

High mobility group box 1 (HMGB1) drives pathologic inflammation by activating signaling through toll-like receptor 4 (TLR4). HMGB1, released from cells either passively (i.e., during necrosis) or actively (i.e., via secretion), triggers signaling through numerous receptors to regulate cytokine production and aggravate lung damage (5,6). Pulmonary edema is caused by AQP 5 by reducing the transcellular rate of the removal of excess water, which then infiltrates alveolar and interstitial spaces (7). AQP 5 plays a crucial role in the nuclear factor-κB (NF-κB) signaling pathway (8). MPO, a marker of neutrophil infiltration in lung tissues, can exacerbate lung pathology by destroying connective tissue and degrading endothelial cell matrix heparan sulfate proteoglycan (9). The first-generation HIV protease inhibitor, saquinavir (SQV), is a potential inhibitor of NF-κB activation and HMGB1 expression (10). Glucocorticoid receptor alpha (GRα), the primary bioactive isoform of the receptor, is normally studied in detail when examining the impact of glucocorticoids. The main reason for the down-regulation of glucocorticoid receptor levels is prolonged glucocorticoid treatment, which affects the treatment of lung inflammation (11). In this study, we investigated the effect of SQV in experimental ALI caused by lipopolysaccharide (LPS) plus methylprednisolone (MPS) *in vitro* and *in vivo*, and identified the mechanism.

## Material and Methods

### Chemicals

SQV (No. 149845-06-7, molecular formula: C_39_H_54_N_6_O_8_S, purity >99%) was provided by Wuhan 3B Scientific Corporation, (China). LPS (L2880, purity >98.0%) was provided by Sigma (China)

### Cell culture

Human pulmonary microvascular endothelial cells (HPMECs, CC-2527) and human type I alveolar epithelial cells (AT I) were purchased from the cell bank of the Chinese Academy of Sciences. Alveolar macrophages (CCL-246) were purchased from the cell bank of ATCC.

### HMGB1, TLR4, phospho-NF-κB (p-NF-κB), and VE-cadherin expression in LPS-stimulated HPMEC cells

HPMECs were maintained in Dulbecco's modified Eagle's medium (DMEM)/F12 containing 10% (v/v) fetal bovine serum at 37°C under a humidified atmosphere of 5% CO_2_ and 95% air. The cells were cultured to approximately 80% confluency in DMEM and were treated with LPS (2 μg/mL) plus SQV at 3 μM or 10 μM with or without the TLR4 inhibitor TAK-242 at 1 μM for 24 h. Then cells were collected and lysed to analyze HMGB1, TLR4, p-NF-κB, and VE-cadherin expression by western blot.

### HMGB1 siRNA transfections

HPMECs were seeded in 6-well plates at a density of 3×10^5^ cells per well in 2 mL antibiotic-free M199 medium with 10% FBS added. HPMECs at 80% confluence were transfected with HMGB1 siRNA transfection reagent according to the manufacturer's instructions. At 24 h post-transfection, LPS and SQV groups were incubated for another 24 h, and then the cells were collected and washed for western blot evaluation. The silencing efficiency was first evaluated with HMGB1 siRNA or HMGB1 siRNA plus 3 μM SQV.

### HMGB1, TLR4, and p-NF-κB expression in LPS-stimulated alveolar macrophage cells

Alveolar macrophage cells (AMs) were maintained in DMEM/F12 containing 10% (v/v) fetal bovine serum at 37°C under a humidified atmosphere of 5% CO_2_ and 95% air. The cells were cultured to approximately 80% confluency in DMEM, then treated with SQV at 3 μM or 10 μM plus LPS (2 μg/ml) for 24 h. Then cells were collected and lysed to analyze HMGB1, TLR4, and p-NF-κB expression by western blot.

### Glucocorticoid receptor alpha (GRα) and IκBα expression in LPS-stimulated HPMECs with methylprednisolone

HPMECs were maintained in DMEM/F12 containing 10% (v/v) fetal bovine serum, 100 kU/L penicillin, and 100 mg/L streptomycin at 37°C in a humidified 5% CO_2_ atmosphere. MPS was administered at 30 μM with or without SQV at 3 μM plus LPS at 2 μg/mL for 96 h, and then cells were collected and lysed with total protein extraction reagent to analyze GRα (Abcam, ab-3580, USA) and IκBα (Abcam, ab-7217) levels.

### Aquaporin 5 (AQP 5), HMGB1, and p-NF-κB expression in AT I cells

AT I cells were maintained in DMEM/F12 containing 10% (v/v) fetal bovine serum at 37°C under a humidified atmosphere of 5% CO_2_ and 95% air. The cells were cultured to 80% confluency in DMEM, then treated with SQV at 3 μM and 10 μM plus LPS (2 μg/mL) for 24 h. Then, cells were collected and lysed to analyze AQP 5, HMGB1, and p-NF-κB expression by western blot.

### Western blot analyses

HPMECs, AMs, and AT I were cultured for 24 h, washed twice with ice-cold phosphate buffered saline (PBS), and lysed in NP40 lysis buffer. The protein concentrations of samples were assayed by a BCA protein assay kit, and equal amounts of cell proteins (50 μg) were separated by SDS-PAGE and analyzed with antibodies specific to HMGB1, TLR4, p-NF-κB, VE-cadherin, AQP 5, and GAPDH. Data were normalized against that of the corresponding GAPDH bands, which were scanned and quantified with the Gel Doc 2000 system (Bio-Rad Laboratories Ltd, USA). The results are reported as fold increases over the control.

### Animals

Adult male Sprague-Dawley (SD) rats (180–200 g body weight) were housed in individual cages under constant temperature (22±2°C) with free access to water and laboratory chow. All animal experimental procedures were performed in accordance with the Institutional Animal Care guidelines and the National Institutes of Health Guide for the Care and Use of Laboratory Animals (USA). The protocol was approved by the Committee on the Ethics of Animal Experiments of Binzhou Medical University (Permit Number: SCXK 20130005).

### LPS-induced two-hit ALI model study protocol

Seventy-five SD rats with an average weight of 180 g were acclimatized for 7 continuous days. A two-hit ALI model was established excluding the sham animals, which was carried out according to previous procedures (12). In brief, an intraperitoneal injection of 2 mg/kg LPS (Sigma Aldrich, USA) with 0.5 mL of saline was administered to each rat. Sixteen hours later, rats were induced by an intratracheal instillation of 4 mg/kg LPS in 0.2 mL of saline. Ten sham rats received an equal volume of saline.

A pilot study was conducted with 3 varying amounts of SQV (50, 100, or 200 mg/kg) to determine the dose-dependent effect in the two-hit ALI rats. SQV post-treatment at doses of 100 and 200 mg/kg significantly lowered the lung index in the two-hit ALI rats (P<0.05). In addition, the recommended dose of SQV for humans is 1800 mg taken orally once daily. According to the body surface area, this dose in rats would be 200 mg/kg, thus, SQV 200 mg/kg was chosen for this study.

The two-hit ALI rats were randomly divided into four groups according to body weight: lung injury group, lung injury plus SQV-treated (200 mg/kg) group, lung injury plus SQV-treated (200 mg/kg) + MPS (20 mg/kg) group, and lung injury plus MPS (20 mg/kg) group. SQV (200 mg/kg) and MPS (20 mg/kg) were given 24 h after LPS administration by intragastric administration and intramuscular administration, respectively, once daily for 5 consecutive days. Five days later, the rats were sacrificed via an intraperitoneal injection of 120 mg/kg pentobarbital. The lungs were excised from the rats by opening the chest via median sternotomy and were used to measure the lung index. Then, the left lobe was removed for the extraction of total protein, and the right lobe was removed and submersed in 10% buffered formalin for 24 h.

### Evans blue vascular permeability assay

Evans blue dye (EBD, 40 mg/kg, Sigma Aldrich Co.) was flushed through the lungs during the last 30 min of *ex vivo* lung perfusion (EVLP) to evaluate pulmonary vascular permeability. After EVLP, lungs were harvested and homogenized in PBS. Lung homogenates were analyzed by measuring absorbance values at 620 nm. Evans blue was gauged as micrograms of dye per gram of wet weight.

### Measurement of myeloperoxidase activity in lung tissues

The myeloperoxidase contents of lung homogenates were measured in duplicate using a commercial kit (Nanjing Jiancheng Bioengineering Institute, China) according to the manufacturer's protocols.

### Western blot analysis of lung tissue

The left lung tissues of rats were homogenized to extract proteins. Protein concentrations of extracts were determined by BCA assay. Equal portions of proteins (50 µg) were portioned by SDS-PAGE and evaluated by western blot with specific antibodies to HMGB1, TLR4, p-NF-κB, VE-cadherin, GRα, IκBα, and GAPDH, and are reported as fold increases over sham animals.

### Histopathological examination

Specimens were processed for paraffin embedding and sectioning. Formalin-fixed, paraffin-embedded sections were cut at a 4.5 mm thickness. Histological sections were loaded onto polylysine slides, de-paraffinized in xylene, rehydrated in graded ethanol dilutions and distilled H_2_O, and then stained with hematoxylin and eosin (HE). A minimum of 20 random high-power fields (400× magnification) were separately scored in a blinded manner. The five histological results were scored with a three-tiered scheme.

### Immunohistochemistry

Immunohistochemistry was performed to detect AQP 5 and p-NF-κB expression in lung tissues. AQP 5 is a specific transmembrane protein responsible for water transport and is expressed in type I lung epithelial cells (13). An automatic immunohistochemistry system, Leica BOND-MAX, was used in this study. Rabbit polyclonal antibodies against AQP 5 (ab104751; Abcam, USA) and p-NF-κB (3033; Cell Signaling, USA) were used at a dilution of 1:1000 and stained with the Leica BOND-MAX system. A DAB enhancer (Leica Microsytems, UK) was applied to increase the intensity of DAB staining. Finally, the samples were analyzed with light microscopy.

### Statistical analysis

Histopathological scores between groups were evaluated using the Wilcoxon rank-sum test. Quantitative data from experiments are reported as means±SD. All statistical tests were conducted using 2-sided tests, and the level of significance was set at P≤0.05.

## Results

### Consequences of SQV on HMGB1 and TLR4 intensity and p-NF-κB activation in HPMECs by LPS stimulation

**Figure 1.**
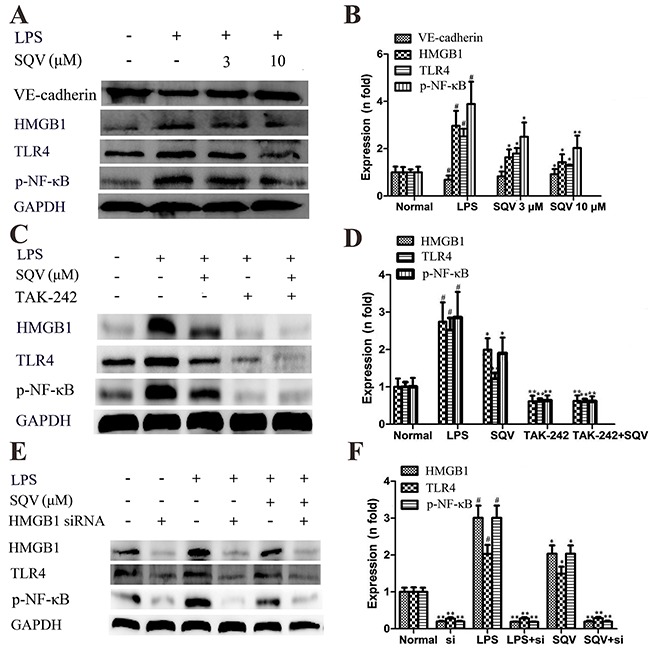
Effects of saquinavir (SQV) on high mobility group box 1 (HMGB1), toll-like receptor 4 (TLR4), and phospho-NF-κB (p-NF-κB) intensity in lipopolysaccharide (LPS)-prompted HPMECs by western blotting. *A* and *B*: human pulmonary microvascular endothelial cells (HPMECs) were incubated with or without LPS for 24 h. *C* and *D*: HPMECs were incubated with LPS with or without the TLR4 inhibitor (TAK-242) for 24 h. *E* and *F*: HPMECs were incubated with LPS with or without HMGB1 siRNA for 24 h. Data are reported as % above normal and means±SD (n=5). ^#^P<0.01 *vs* the normal group; *P<0.05, **P<0.01 *vs* the LPS group (ANOVA with Dunnett's test).

HMGB1 was activated by LPS, increasing the expression of HMGB1, TLR4, and p-NF-κB, which is downstream of HMGB1, in HPMECs upon 24 h treatment. VE-cadherin has a critical role in the maintenance of vascular integrity and organization of the endothelial cell cytoskeleton (14). LPS remarkably reduced VE-cadherin expression in HPMECs upon 24 h treatment. However, pretreatment of HPMECs with SQV significantly down-regulated the production of LPS-induced HMGB1 and TLR4, and reduced p-NF-κB triggering, weakening the decrease of VE-cadherin (Figure 1A and B).

To explore the mechanism of LPS in HMGB1-TLR4 activation, the TLR4 inhibitor TAK-242 (resatorvid) was used to inhibit TLR4 signaling in HPMECs. The results demonstrated that TLR4 expression was decreased in LPS-stimulated HPMECs using TAK-242. Nevertheless, HMGB1 expression was decreased slightly in LPS-stimulated HPMECs with TAK-242. LPS-stimulated HPMECs using TAK-242 plus SQV did not reduce p-NF-κB compared with LPS-stimulated HPMECs using TAK-242 (Figure 1C and D).

To determine whether HMGB1 is involved in the LPS-induced production of HMGB1, TLR4, and p-NF-κB in HPMEC cells, the expression of HMGB1 was silenced in HPMEC cells by stable transfection of a vector expressing HMGB1 siRNA. The result demonstrated that HMGB1, TLR4, and p-NF-κB expression were reduced in LPS-stimulated HPMECs with HMGB1 siRNA. LPS-stimulated HPMECs with HMGB1 siRNA plus SQV did not reduce HMGB1, TLR4, or p-NF-κB compared to LPS-stimulated HPMECs with HMGB1 siRNA (Figure 1E and F). The results suggested that SQV inhibited inflammation via blocking the TLR4/NF-κB pathway.

### Effects of SQV on HMGB1, TLR4, and p-NF-κB expression in AMs by LPS stimulation

**Figure 2.**
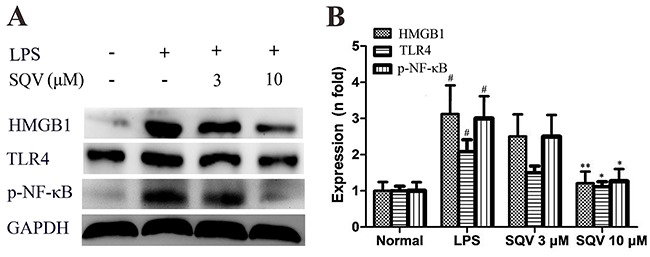
Effects of saquinavir (SQV) on high mobility group box 1 (HMGB1), toll-like receptor 4 (TLR4), and phospho-NF-κB (p-NF-κB) intensity in lipopolysaccharide (LPS)-prompted alveolar macrophages by western blotting. Data are reported as % above normal and means±SD (n=5). ^#^P<0.01 *vs* the normal group. *P<0.05, **P<0.01 *vs* the LPS group (ANOVA with Dunnett's test).

AMs play an important role in the development of acute inflammatory lung injury (15). When AMs were incubated with SQV combined with a stimulatory dose of LPS, significant reductions of HMGB1, TLR4, and p-NF-κB were observed compared to LPS alone. This suggested that SQV down-regulated the LPS-induced production of inflammation-associated proteins in AMs (Figure 2).

### Effects of SQV on GRα and IκBα expression in LPS-stimulated HPMECs with methylprednisolone

**Figure 3.**
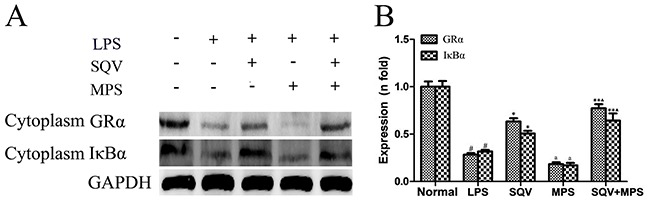
Effects of saquinavir (SQV) on glucocorticoid receptor alpha (GRα) and IκBα expression in lipopolysaccharide (LPS)-stimulated human pulmonary microvascular endothelial cells by western blotting. MPS: methylprednisolone. Data are reported as means±SD (n=5). ^#^P<0.01 *vs* the normal group; *P<0.05, **P<0.01, ^a^P<0.05 *vs* the LPS group; ^▾^P<0.05 *vs* the MPS group (Dunnett's test).

A large reduction of GRα in the cytoplasm is a main cause of GRα deficiency or glucocorticoid resistance. In this study, decreased GRα and IκBα levels in the cytoplasm of HPMECs were noted with LPS stimulation plus MPS at 30 μM for 96 h. This showed that GRα deficiency or glucocorticoid resistance was present (Figure 3). However, LPS-stimulated HPMECs with SQV at 3 μM had significantly attenuated decreases of GRα and IκBα in the cytoplasm with MPS at 30 μM and sustained LPS stimulation for 96 h. Notably, these cells avoided GRα deficiency compared with those treated with MPS at 30 μM for 96 h as shown in Figure 3. These results suggested that SQV increased GRα expression in the cytoplasm in LPS plus MPS-stimulated HPMECs, which prevented GRα deficiency.

### Effects of SQV on AQP 5, HMGB1, and p-NF-κB expression in LPS-stimulated AT I cells

**Figure 4.**
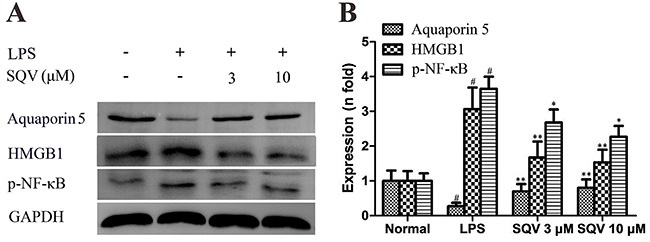
Influence of saquinavir (SQV) on high mobility group box 1 (HMGB1), phospho-NF-κB (p-NF-κB), and aquaporin 5 in lipopolysaccharide (LPS)-stimulated human type I alveolar epithelial cells (AT I) by western blotting. Data are reported as means±SD (n=5). ^#^P<0.01 *vs* the normal group; *P<0.05, **P<0.01 *vs* the LPS group (ANOVA with Dunnett's test).

In LPS-stimulated AT I cells, SQV strongly inhibited HMGB1 and p-NF-κB expressions and increased AQP 5 in a concentration-dependent manner. These results indicated that SQV reduced AT I injury with higher AQP 5 levels and lower HMGB1 and p-NF-κB expression (Figure 4).

### Effects of SQV on pulmonary capillary permeability

**Figure 5.**
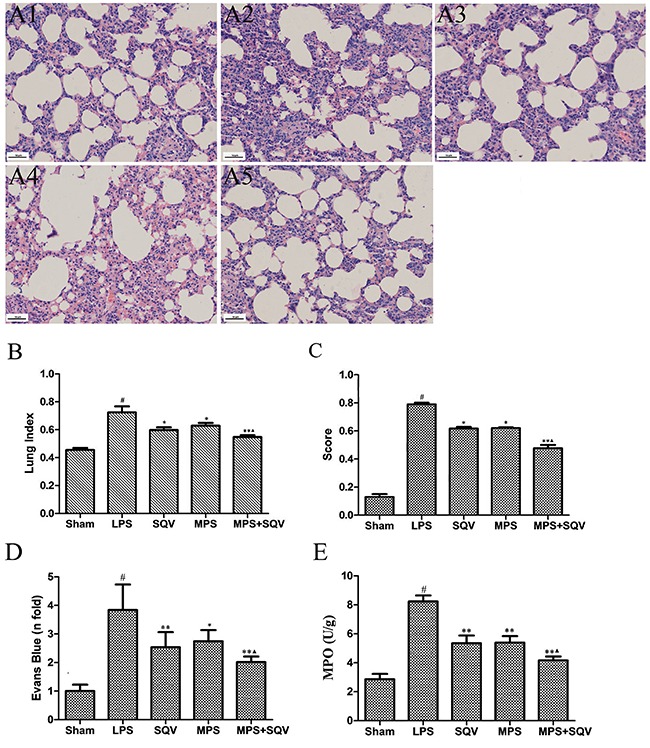
Influence of saquinavir (SQV) plus methylprednisolone (MPS) on histopathological change and lung index in acute lung injury (ALI) model. Typical light minute appearance of hematoxylin and eosin staining for sham (*A1*), lipopolysaccharide (LPS) (*A2*), SQV (*A3*), MPS (*A4*), and MPS plus SQV (*A5*). Scale bar = 50 µm. *B* and *C*: Effects of SQV on the lung index and scores of histopathological change in the two-hit ALI rats. *D*: Effects of SQV on pulmonary capillary permeability. *E*: Effects of SQV on MPO activity in lung tissue. Data are reported as means±SD. ^#^P<0.01 *vs* the sham group; *P<0.05, **P<0.01 *vs* the LPS-treated rats. ^▾^P<0.05 *vs* the MPS group. Histopathological scores between groups were evaluated using the Wilcoxon rank-sum test. Quantitative data was analyzed by ANOVA with Dunnett's test.

EBD content in bronchoalveolar lavage fluid was used to analyze pulmonary capillary permeability. Compared to the sham group, the EBD content was significantly increased in the LPS group (P<0.01), which was significantly decreased after SQV treatment (P<0.01) (Figure 5D). In addition, the EBD content in SQV plus MPS-treated animals was lower than in rats treated by MPS only (P<0.05) (Figure 5D). These results suggested that SQV retains its vascular barrier-protective effects in LPS-induced ALI with glucocorticoid insensitivity.

### Effects of SQV on MPO activity in lung tissue

LPS significantly increased the MPO activity in rats compared with that of the sham group (P<0.01). SQV and MPS treatment remarkably decreased MPO activity (P<0.01). In addition, SQV plus MPS treatment resulted in lower MPO activity than in rats treated only with MPS (Figure 5E).

### Effects of SQV on histopathology and lung index *in vivo*

In this study, a clinically relevant two-hit rat model of LPS was utilized to further assess the efficacy of SQV in ALI. Rats were challenged with LPS (2 mg/kg). Histological assessment of HE-stained lung sections from multiple independent animals demonstrated decreased edema formation, neutrophil invasion, and disruption of lung tissue morphology in rats receiving SQV (Figure 5A). These results suggested that SQV retained its barrier-protective and anti-inflammatory effects in LPS-induced ALI with glucocorticoid insensitivity. The lung weight to body weight ratio (lung index), a measure of pulmonary edema, was used to assess the breakdown of the alveolar-capillary barrier in ALI with glucocorticoid insensitivity. Two-hit-induced ALI with glucocorticoid insensitivity led to a higher lung index compared to the control animals, and SQV attenuated this increase. This clearly demonstrated that SQV was effective in LPS-reduced glucocorticoid insensitivity, leading to a higher lung index as shown in Figure 5B.

**Figure 6.**
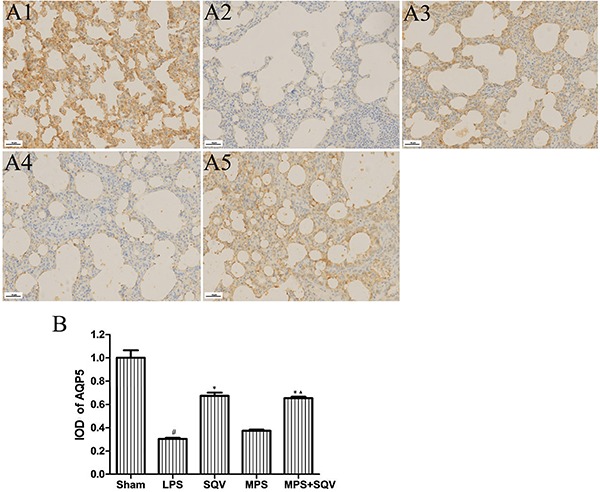
Influence of saquinavir (SQV) plus methylprednisolone (MPS) on the expression of aquaporin 5 (AQP 5) in acute lung injury (ALI) model. Immunohistochemistry of AQP 5 for sham (*A1*), lipopolysaccharide (LPS) (*A2*), SQV (*A3*), MPS (*A4*), and MPS plus SQV (*A5*). Scale bar = 50 µm. *B*, Effects of SQV on the integral optical density of AQP 5 in the two-hit ALI rats (n=5). Data are reported as means±SD. ^#^P<0.01 *vs* the sham group; *P<0.01 *vs* the LPS-treated rats. ^▾^P<0.01 *vs* MPS group (Dunnett's test).

AQP 5 was stained brown largely within the cytoplasm of normal lung samples by immunohistochemistry as revealed in sham animals shown in Figure 6 A1. However, lower AQP 5 was seen in two-hit-induced ALI animals (Figure 6 A2). The SQV plus MPS-treated animals showed larger amounts of AQP 5 in the lung than in the two-hit-induced ALI animals by immunohistochemistry staining (Figure 6 A5).

### Effects of SQV on NF-κB activation and TLR4, HMGB1, GRα, and IκBα expressions in lung tissues of experimental ALI rats

**Figure 7.**
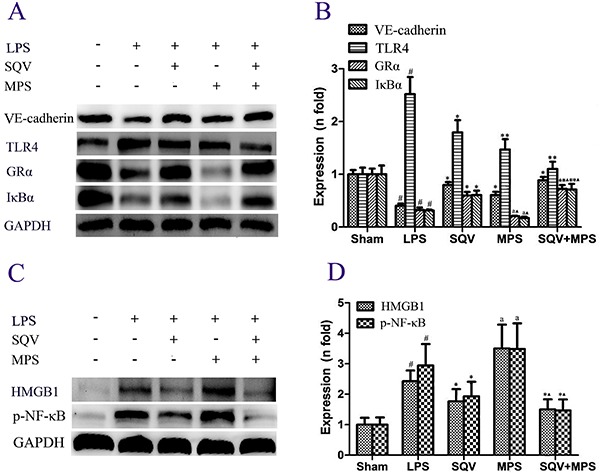
Influence of saquinavir (SQV) plus methylprednisolone (MPS) on high mobility group box 1 (HMGB1), toll-like receptor 4 (TLR4), glucocorticoid receptor alpha (GRα), IκBα, VE-cadherin, and phospho-NF-κB (p-NF-κB) expression in acute lung injury (ALI) rats. LPS, lipopolysaccharide. Data are reported as means±SD. ^#^P<0.01 *vs* the sham group; *P<0.05, **P<0.01, ^a^P<0.05 *vs* the LPS-treated rats. ^▾^P<0.05 *vs* the MPS group (ANOVA with Dunnett's test).

**Figure 8.**
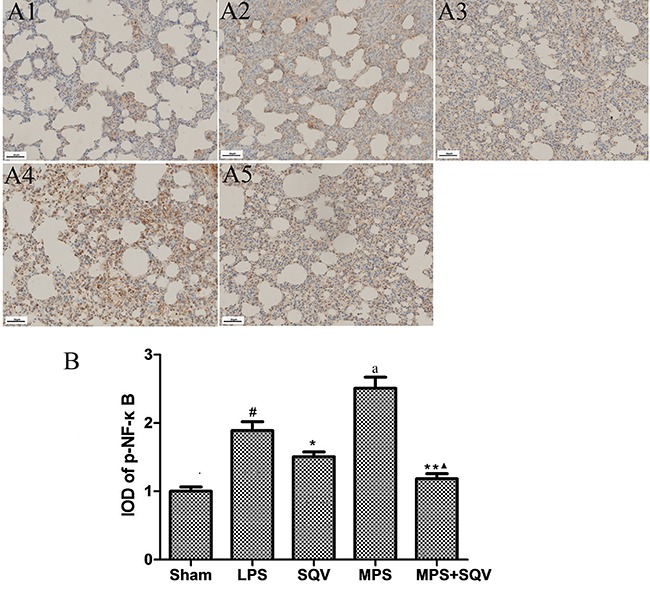
Influence of saquinavir (SQV) plus methylprednisolone (MPS) on the expression of phospho-NF-κB (p-NF-κB) in acute lung injury (ALI) model. *A*: Immunohistochemistry of p-NF-κB for sham (*A1*), lipopolysaccharide (LPS) (*A2*), SQV (*A3*), MPS (*A4*), and MPS plus SQV (*A5*) Scale bar = 50 µm. *B*: Effects of SQV on the integral optical density of p-NF-κB in the two-hit ALI rats (n=5). Data are reported as means±SD. ^#^P<0.01 *vs* the sham group; *P<0.05, **P<0.01, ^a^P<0.01 *vs* the LPS-treated rats. ^▾^P<0.01 *vs* the MPS group (ANOVA with Dunnett's test).

As expected, rats exposed to the two-hit model with glucocorticoid insensitivity displayed increased HMGB1, TLR4, and p-NF-κB, and decreased VE-cadherin, GRα, and IκBα protein expression, which is indicative of severe lung injury. These abnormal conditions were mitigated dramatically in animals receiving SQV. This revealed that SQV was protective in this model and relieved glucocorticoid insensitivity to a large degree, as shown in Figure 7. NF-κB plays a pivotal role in inflammatory and immune responses, and more p-NF-κB was expressed in LPS-induced ALI rats (Figure 8 A2). SQV decreased the expression of p-NF-κB (Figure 8 A3), and SQV plus MPS-treated animals had less p-NF-κB in the lungs than MPS-induced ALI animals according to immunohistochemical staining (Figure 8 A4 and A5).

## Discussion

ARDS is a syndrome characterized by hypoxemia and respiratory insufficiency. A number of common pathological pulmonary features appear in ARDS patients, such as increased permeability as reflected by alveolar edema due to epithelial and endothelial cell damage, and neutrophil infiltration in the early phase (3). There are very few effective therapies for ARDS other than the use of lung-protective ventilation and conservative fluid management (16,17). Glucocorticoids have proven to be remarkably successful drugs in the treatment of chronic inflammatory diseases, such as asthma, rheumatoid arthritis, and inflammatory bowel disease. However, some patients with moderate to severe disease show partial or complete resistance to the anti-inflammatory actions of glucocorticoids. Therefore, novel therapeutics must be exploited to ameliorate inflammation and glucocorticoid insensitivity in ALI and ARDS patients (18).

The pathogenic basis of ALI or ARDS and factors governing susceptibility are incompletely understood. Different animal models of experimental lung injury have been used to investigate the mechanisms of lung injury. LPS, the main component of the outer wall of gram-negative bacteria, is known to activate the inflammatory cascade, resulting in the release of inflammatory mediators that induce the development of ALI and ARDS, so LPS administration is the most often used approach to model the consequences of bacterial ARDS. The LPS two-hit ALI model has been shown to induce broad inflammatory reactions and the resulting damage, and is more consistent, reliable, and directly related to diagnostic criteria. It more closely imitates the pathological sequence of ALI due to extended hypoxemia and certain forms of pulmonary damage in rats. We chose to intraperitoneally inject LPS at 2 mg/kg, then performed tracheal infusion using LPS at 4 mg/kg to create a two-hit model. This resulted in lung injury in the model, including more neutrophils within the alveolar and the interstitial areas, and proteinaceous refuse covering the airspaces and alveolar septum. The two-hit model was administered with SQV and MPS once daily for 5 consecutive days 24-h post-LPS. Post-LPS rescue therapy may be more clinically relevant. Our results revealed that treatment with SQV for 3 continuous days reduced histopathological injury, including fewer neutrophils in the alveolar and interstitial spaces, less proteinaceous refuse covering the airspaces, and thinner alveolar septa. This suggested that continuous treatment with SQV can attenuate histopathological injury to ease ALI.

The breaching of microvascular walls culminates in extravascular aggregation of edema fluid full of protein, which is the pivotal pathophysiological mechanism in acute lung damage. Elevated permeability is tied to the movement of leukocytes and erythrocytes to the alveolar area in ARDS. VE-cadherin perpetuates endothelial barrier disruption in lung microvessels; interruption of VE-cadherin homophilic interactions minimizes lung microvascular blockage (19). Lung and body-wide endothelial impediments are critical therapeutic targets in ARDS (20). Our results showed that treatment with SQV attenuated the decrease of VE-cadherin in LPS-stimulated HPMECs *in vitro* and in the LPS-induced two-hit model *in vivo*. In addition, treatment with SQV decreased the lung index and MPO activity and improved pulmonary capillary permeability. This suggested that SQV can maintain pulmonary microvascular endothelial cell integrity against LPS *in vitro* and *in vivo*.

Macrophages are essential to innate immunity and host defense, play an important role in the lung and alveolar space, and macrophage activation and chemokine production are key events in the development of acute lung injury (21,22,14). Our results showed that treatment with SQV inhibited secretion of HMGB1 and p-NF-κB in LPS-stimulated AMs. This suggested that SQV can inactivate LPS-stimulated AMs *in vitro*.

AT I cells are squamous and cover 90–95% of the alveolar surface. Type I cells are involved in the process of gas exchange between the alveoli and blood. AQP 5 is at the apical membrane of AT I to drive water transit between alveolar epithelium and capillary alcoves (23). LPS activates alveolar epithelium disruption and swelling. In the present study, SQV ameliorated the LPS-induced elimination of AQP 5 in AT I cells. AQP 5 expression was distinctly shown by immunohistochemistry, revealing that SQV inhibited AQP 5 downregulation in LPS-stimulated AT I cells *in vitro*.

HMGB1 is a central factor in initiating and maintaining the inflammation cascade, and thus plays a crucial role in ARDS (24,25). HMGB1 is also an independent biomarker of ICU mortality in patients with ARDS (26). Based on these observations, we evaluated the effect of SQV on the production of inflammatory mediators in ALI rats. NF-κB activation is triggered in lung macrophages of ARDS patients, and in human podocytes cultured with TNF-α and LPS, SQV significantly blunts NF-κB up-regulation (27,28). Furthermore, treatment with SQV inhibits LPS-induced NF-κB activation as well (29). In this study, treatment with SQV suppressed the expression of p-NF-κB caused by LPS and MPS.

HMGB1 binds with TLR4 and acquires intracellular and extracellular stimulation through NF-κB activation (30–32). HMGB1/TLR4/NF-κB signaling thus plays a key part in the development of ARDS. Inhibition of HMGB1 expression or blockage with an anti-HMGB1 antibody prevents or minimizes ARDS (33,34). Previous studies have shown that SQV limits the TLR4-mediated inflammatory pathway, reduces NF-κB activation, and reduces HMGB1 expression from ventilator-induced lung injury in mice (9). Our study showed that treatment with SQV ameliorated lung damage with lower HMGB1 and p-NF-κB expression in lung tissues of LPS two-hit ALI rats. Furthermore, we revealed that SQV inhibited the secretion of HMGB1 and p-NF-κB in LPS-stimulated HPMECs *in vitro*. These results suggested that SQV ameliorated lung damage by modulating HMGB1/TLR4/NF-κB signaling.

SQV has been on the market for several years, its safety profile is well known, and it is easy and inexpensive to obtain for clinical trials. It has many advantages, including that phase I clinical studies can be exempted, and nonclinical safety data is available in the public domain, such as for chronic toxicity, genotoxicity, reproductive effects, development toxicity, and carcinogenicity. All told, drug repositioning reduces the cost and risk of R&D. NF-κB and GRα mediate the regulation of systemic and pulmonary inflammation during ARDS, and GC-GRα down-regulation of NF-κB activation is critical for the resolution of systemic and pulmonary inflammation in ARDS (35). Methylprednisolone (1 mg/kg/d) down-regulation of systemic inflammation is associated with significant improvement in pulmonary and extra-pulmonary organ dysfunction and a reduction in the duration of mechanical ventilation and ICU length of stay (36). However, glucocorticoid resistance or GRα deficiency with lower GRα expression in the cytoplasm is a general phenomenon in systemic inflammation when a high dose of glucocorticoids is used for long time, especially in unresolved ARDS. Two cellular signaling pathways are central to the regulation of inflammation in ARDS with lower GRα expression in the cytoplasm, the stimulatory NF-κB and the inhibitory GRα (37). NF-κB and GRα have diametrically opposed functions in regulating inflammation. Knowing how to attenuate the decrease of GRα and IκBα in the cytoplasm, or reduce the decrease of p-NF-κB in the nucleus to avoid GRα deficiency is very important. How to stimulate GRα deficiency and even glucocorticoid tolerance *in vitro* is not well documented. Our study showed that LPS plus MPS at 30 μM decreased GRα and IκBα largely in the cytoplasm to model GRα deficiency and glucocorticoid tolerance in HPMECs. In addition, SQV plus MPS attenuated the decrease of GRα and IκBα in the cytoplasm of LPS-stimulated HPMECs compared to MPS alone. Similar results were shown in LPS plus high dose MPS-induced ALI rats, which resulted in lower GRα expression in the cytoplasm.

These results suggested that SQV plus glucocorticoids can resolve systemic inflammation to a large degree by inhibiting the decrease of GRα and IκBα in the cytoplasm, thus improving glucocorticoid resistance as well. In addition, SQV can reduce the time-course and dose of glucocorticoids used in ALI and ARDS. That is, SQV supports the anti-inflammatory effects of glucocorticoids and largely avoids GRα deficiency, reducing adverse reactions. In follow-up studies, we will investigate the long-term protective effects of SQV on lung function to see if it can delay and diminish pulmonary fibrosis.
